# Acute pancreatitis associated with everolimus after kidney transplantation: a case report

**DOI:** 10.1186/s12882-016-0376-6

**Published:** 2016-10-28

**Authors:** Francesco Fontana, Gianni Cappelli

**Affiliations:** Surgical, Medical and Dental Department of Morphological Sciences, Section of Nephrology, University of Modena and Reggio Emilia, Modena, Italy

**Keywords:** Everolimus, Acute pancreatitis, Kidney transplantation, Case report

## Abstract

**Background:**

Acute pancreatitis (AP) following KT is a rare and often fatal complication of the early post-transplant period. Common causative factors for AP are rare after KT; anti-rejection drugs as CyA, prednisone and MMF have been implicated, although evidence is not strong and we found no reports on possible causative role for mTOR inhibitors.

**Case presentation:**

A 55-year-old Caucasian man with end-stage renal disease due to idiopathic membrano-prolipherative glomerulonephritis underwent single kidney transplantation (KT) from cadaveric donor. Anti-rejection protocol was based on Basiliximab induction followed by prednisone and mycophenolate mophetil (MMF) and Cyclosporine; Everolimus (Eve) was scheduled to substitute MMF at week 3. At day 1 he had an asymptomatic elevation of pancreatic enzymes, spontaneously resolved. The further course was unremarkable and on day 19 he started Eve, with following asymptomatic rise in pancreatic enzymes. At day 33 the patient presented with abdominal pain and a marked elevation in serum amylase (1383 U/l) and lipase (1015 U/l), normal liver enzymes and bilirubin, no hypercalcemia, mild elevation in triglycerids; RT-PCRs for Cytomegalovirus or Epstein-Barr virus were negative. The patient had no history of alcohol abuse; ultrasound, CT and MRI found no evidence of biliary lithiasis. CT scans showed a patchy fluid collection in the pancreatic head area, consistent with idiopathic necrotizing pancreatitis. The patient was treated medically and Eve was withdrawn 1 week after. Patient underwent guided drainage of the fluid collection, but developed bacterial sepsis; surgical intervention was required with debridement of necrotic tissue, lavage and drainage; immunosuppression was totally withdrawn. Following course was complicated with multiple systemic infection. Transplantectomy for acute rejection was performed, and patient entered hemodialysis.

**Conclusions:**

Our patient had a presentation that is consistent for a causative role of Eve. A predisposing condition (acute pancreatic insult during transplant surgery) spontaneously resolved, relapsed and evolved rapidly in AP after the initiation of treatment with Eve with a consistent time latency. None of the well-known common causative factors for AP was present. We discourage the use of Eve in patients with recent episodes of sub-clinical pancreatitis, since it may represent a precipitating factor or interfere with resolution.

## Background

Since its first description by Starlz in 1964 [1], acute pancreatitis (AP) following kidney transplantation (KT) has been recognized as a rare and often fatal complication of the early post-transplant period, with incidence rates reported ranging from 1 to 7 %, and an extremely high mortality rate (from 60 to 100 %) [2–4]. AP in the post-transplant patient represents a more complex clinical challenge with respect to the general population: causative factors might be different and often unrecognized, mitigated early clinical symptoms in immunosuppressed patients make early diagnosis and determination of the severity of AP more difficult, and there is no consensus on the more appropriate treatment timing and strategy. With regard to possible etiologies, common causative factors (biliar lithiasis, alcohol abuse) are not frequent in the transplant population; iatrogenic causes have been advocated, and immunosuppressive therapy must be taken into consideration. While there is a definite causative role for Azathioprine and (although less, and especially regarding dexamethasone) for steroids [5, 6], things appear more uncertain for cyclosporine and mycophenolate [7]; we found no reports about supposed causative role for mTOR inhibitors in the pathogenesis of AP after KT.

## Case presentation

A 55-year-old Caucasian man with end-stage renal disease due to idiopathic membrano-prolipherative glomerulonephritis, who had been in chronic renal replacement therapy with hemodialysis for 8 years, underwent single kidney transplantation from cadaveric donor. The patient had a distal abdominal aortic aneurysm corrected with endoprosthesis, and had had a previous surgical correction of a common iliac artery aneurysm (contralateral to the graft positioning); he had no previous history of pancreatitis, gallbladder or biliary lithiasis. He had no family history of pancreatic or biliary disorders.

**Fig. 1:**
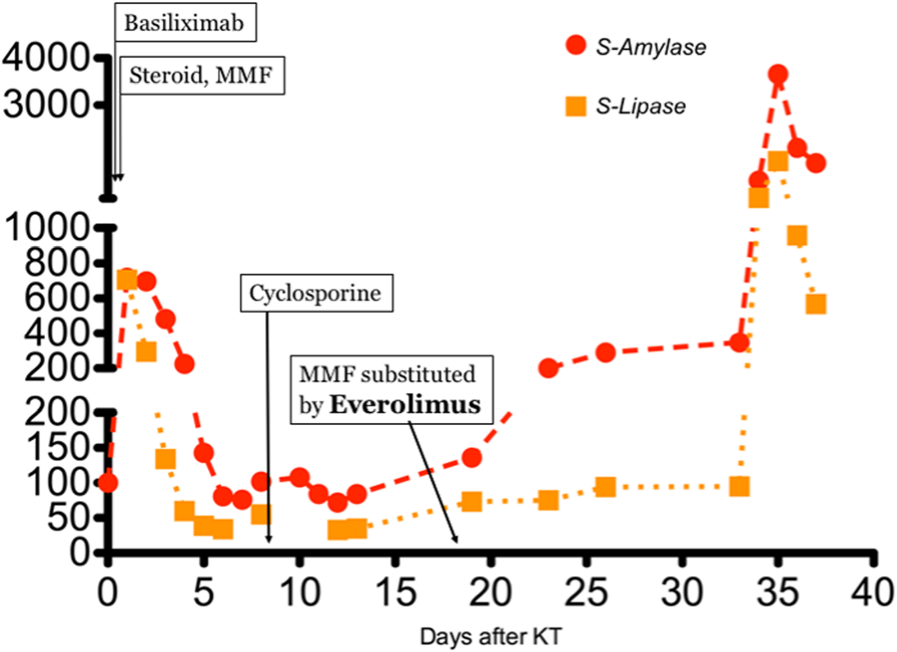
Trends of pancreatic enzymes since KT

**Fig. 2:**
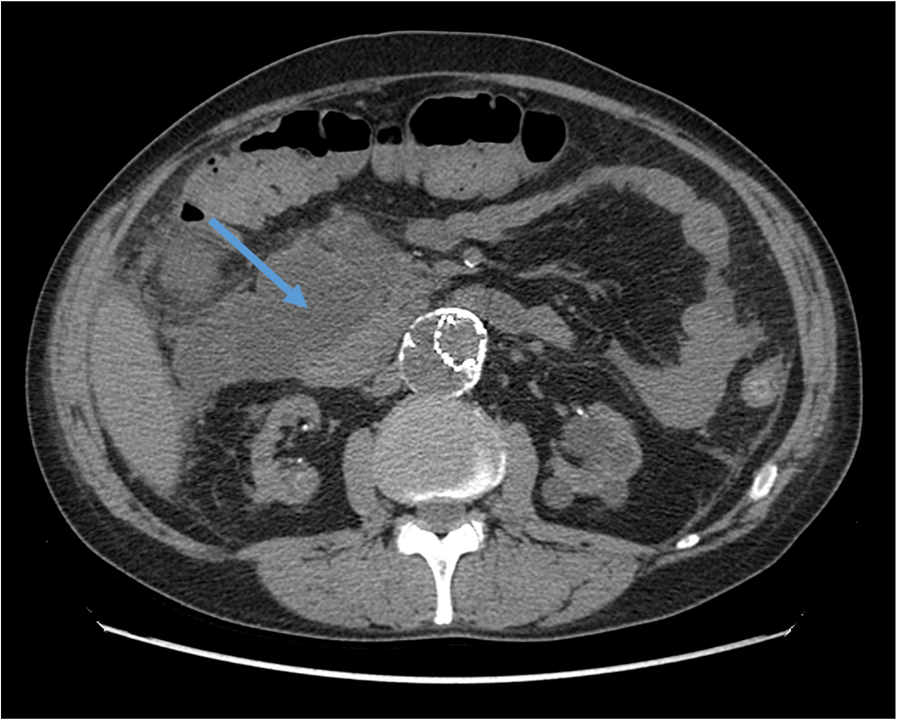
Abdominal CT Scan showing the initial area of patchy necrosis around the head of pancreas

Induction treatment for transplantation consisted in Basiliximab, prednisone and mycophenolate mophetil (MMF); after surgery, he presented delayed graft function that required two consecutive dialytic sessions. Of note, at day 1 after transplant (while anuric) he had an asymptomatic elevation of pancreatic enzymes (peak of serum amylase: 718 U/l), that gradually resolved in 5 days. From day 8 he started receiving cyclosporine. The patient also received anti-CMV prophylaxis with Valaciclovir. The further course was unremarkable, and the patient was regularly discharged at day 14 with a serum creatinine of 2,1 mg/dl. However, 5 days after he presented at follow up visit with colic pain involving the upper right quadrant of the abdomen; an abdominal ultrasonography showed a normally distended gallbladder, with no dilatation of the common bile duct or biliary three; he had no frank elevation of pancreatic enzymes. The patient received a course of antibiotics for evidence of pneumonia at chest X-ray. On that day, he started Everolimus, (the patients was enrolled in a trial that addressed the possibility of minimizing calcineurin inhibitors nephrotoxicity with the use of mTOR inhibitors); the target through-levels for immunouppressors were 8 ng/dl for Everolimus and 300 ng/dl for Cyclosporine. After two more weeks the patients had an episode of diarrhea; MMF was withdrawn (following the study protocol), and Everolimus dose was increased to reach target levels (on that day, blood level was 5,11 ng/ml). The patient had mild elevation in pancreatic enzymes, asymptomatic, since the beginning of treatment with Everolimus (Fig. 1). There was also evidence of mild rise in serum triglycerides (ranging from 240 to 330 mg/dl) with normal total and LDL cholesterol, for which appropriate dietary advice was preferred to lipid-lowering treatment, according to current guidelines [8]. On day 34 after KT, the patient presented to the emergency department with pain at the upper quadrants of the abdomen; he had marked elevation of pancreatic enzymes (serum amylase 1383 U/l, serum lipase 1015 U/l), no elevation in liver enzymes or bilirubin, mild leukocytosis (white blood cells count: 10,13 × 10^3^/ul), no hypercalcemia (serum calcium 8,2 mg/dl), moderate elevation in triglycerides (400 mg/dl); RT-PCRs for Cytomegalovirus or Epstein-Barr virus were negative; at presentation APACHE score II was 10 points, and after 48 h RANSON score was 4. The patient had no history of alcohol abuse; ultrasound, CT and MRI found no evidence of biliary tract or gallbladder lithiasis. CT abdominal scans confirmed the presence of a patchy fluid collection in the pancreatic head area, extending to gastric antrum and duodenum and posteriorly to the right iliopsoas muscle (Fig. 2). A diagnosis of idiopathic necrotizing pancreatitis was made. The patient was treated medically, and immunosuppressive therapy initially maintained (with lowered target levels) in the attempt of protecting graft function; although, considered the scarce improvement, Everolimus was withdrawn 1 week after the beginning of symptoms and mild immunosuppression continued with Cyclosporine and steroid intravenously. Graft function after initial worsening remained stable (creatinine 2,5 mg/dl). After 1 week of unsuccessful medical treatment, the patient underwent CT-guided drainage of the fluid collection in the pancreatic head, procedure that was repeated three times in the first month. The course was complicated by infection of the fluid collection with Staphylococcus Haemolyticus and Staphylococcus Epidermidis, and subsequent development of inflammatory systemic response and sepsis. The patient underwent surgical intervention with debridement of necrotic tissue, lavage and drainage 45 days after the beginning of symptoms; at that point, immunosuppressive treatment was totally omitted. Following course was complicated with multiple systemic infection with Stenotrophomonas Spp, Klebsiella Spp, Pseudomonas Spp, Candida spp which required prolonged combination antibiotic therapy. After one more month of medical care an allograft biopsy was performed for worsening kidney function and acute abdominal pain; histologic examination showed signs of Banff type II acute rejection with diffuse hemorrhagic and infarction areas. Transplantectomy was performed, and patient restarted on hemodialysis. The patient eventually survived infections, and was discharged after 5 months.

## Discussion

AP is a well known complication following KT. Besides traditional etiologies (biliary tract stones, alcohol, hypertriglyceridemia, hypercalcemia), a number of possible contributing factors have been proposed in the renal transplant patient: surgical trauma, corticosteroids (especially pulse therapy), viral infections, immunosuppressive drugs. Although among the causative factors for AP adverse drug reactions are considered to be rare, the exclusion of all the common etiologies imposes this evaluation.

The diagnosis of drug-induced AP is difficoult to establish, mainly due to the absence of cause-specific diagnostic tests, and it is usually based on the following criteria: AP occurring during the administration of a drug and with compatible latency time, exclusion of all other common causes, disappearance of symptoms of AP after drug withdrawal and symptoms recurrence after re-challenge with suspected drug [5, 7].

Many of the commonly used maintenance immunosuppressive medications (azathioprine, cyclosporine, prednisone, MMF) have been implicated clinically and experimentally in the cause of AP. Azathioprine (which is not currently used nowadays) is thought to have the strongest association, and is classified as class I medication (implicated in greater than 20 reported cases of AP with at least one documented case following re-exposure) [5]. Cyclosporine is classified as class III drug (only 2 published case reports giving the dosage and latency are required to be in this category) [6]; nevertheless, one study involving 466 KT recipients showed an apparent proclivity of Cyclosporine-treated patients to develop biliary calculous disease [9]. MMF has only one report for probable causation of AP after KT [7].

We found no reports about the possible causative role of AP after KT for mTOR inhibitors. Nevertheless, Subramaniam et al [10] reported the case of a young man who developed severe hypertriglyceridemia (serum triglycerides greater than 1000 mg/dl) and acute pancreatitis after a 2 months treatment course of everolimus for metastatic pancreatic neuroendocrine tumor; of note, everolimus is signaled to have an experimentally proven synergistic negative effect with Cyclosporine on pancreatic islet dysfunction in rats [11].

When compared with general population, AP after KT has a more serious course; whereas in non-immunosuppressed patients overall mortality is about 5–10 %, in renal transplant patients the average mortality is 61,3 % while in cases of necrotizing AP reaches 100 % [2, 4, 12]. Necrotizing form of AP is more frequent following KT [4, 13]. Although experimental evidence is lacking, diminished immunocompetence and defective macrophage and neutrophil chemotactic and phagocytic function in KT patient may result in an initially attenuated inflammatory response with decreased tissue perfusion and delayed, ineffective clearance of damaged tissue with consequent expansion of extensive necrosis and infection. KT recipients have a more insidious onset of illness with fewer signs and symptoms, and the clinical presentation can be misleading [2, 4].

Our patient had a presentation that was consistent for a causative role of Everolimus for the development of AP. Although there are an obvious number of confounding factor, our patient appeared to have had a predisposing condition (acute pancreatic insult possibly during/after transplant surgery spontaneously resolved and not aggravated by initial immunosuppressive treatment) that relapsed rapidly after the initiation of treatment with everolimus (peaking with its maximum dosage). There was a consistent time latency (14 days) for everolimus as causative agent or at least the last and preponderant precipitating factor. None of the well-known common risk factors for AP was present in our patients neither before neither at the moment of AP presentation: he had no bile tract or gallbladder lithiasis (as shown by repeated CT and MRI exams), no history of alcohol abuse, no hypercalcemia, no active viral infections. He only developed modest hypertriglyceridemia (known complication of mTOR inhibitor therapy), that is not sufficient itself to justify the clinical presentation of acute necrotizing pancreatitis [14]. The history of our patient had a tumultuous course mainly due to the delay in surgical intervention and withdrawal of immunosuppressive treatment, justified by the strenuous effort to save the graft, according to patient’s will. Our supposed association between everolimus treatment and AP lacks the strength of a remission after withdrawal of therapy. This is due in our opinion to two reasons: the lack of recognition of this possible association (never mentioned in literature before) and superimposed infection that lately conditioned the gravity of clinical picture. Obviously, re-challenge with the supposed causative drug was not attempted. Our patient ultimately survived but lost the graft for a non-renal complication and possibly avoidable cause. The patient gave informed consent on the publication of data.

## Conclusions

In conclusion, we present the case of acute necrotizing pancreatitis 1 month after kidney transplantation after the introduction of Everolimus and without evidence of any other common causative factor for AP. Our patient had a previous post-surgical episode of asymptomatic and spontaneously resolved elevation in pancreatic enzymes and enzyme levels slowly started to rise shortly after Everolimus treatment was commenced. We warn clinicians to have a high degree of suspicion for AP in KT transplant patients and possibly avoid mTOR inhibitors in patients who had previous episodes of subclinical pancreatitis, since it may represent a precipitating factor.

## Abbreviations

AP: Acute pancreatitis
KT: Kidney transplantation
MMF: Mycophenolate mophetil
